# Dermoscopy at an Inflection Point: Sharper Criteria, New Optics, and the Arrival of Artificial Intelligence

**DOI:** 10.3390/cancers18203277

**Published:** 2026-10-11

**Authors:** Dana N. Pham, Warren H. Chan, Jade Conway, Iman Ali, Clay J. Cockerell

**Affiliations:** 1 Orlando College of Osteopathic Medicine, Winter Garden, FL 34787, USA; dpham039@students.ocom.org; 2 Reveal Research Institute, Dallas, TX 75235, USA; 3 Texas Health Dallas, Dallas, TX 75231, USA; 4 The Departments of Dermatology and Pathology, The University of Texas Southwestern Medical Center, Dallas, TX 75390, USA; 5 Lake Granbury Medical Center, Dallas, TX 75235, USA

**Keywords:** dermoscopy, teledermoscopy, melanoma, basal cell carcinoma, squamous cell carcinoma, reflectance confocal microscopy, optical coherence tomography, artificial intelligence

## Abstract

Dermoscopy improves the diagnosis of melanoma and keratinocyte carcinoma, but its value depends on the observer, the lesion mix, the anatomic site, and the action taken after the examination. Recent reviews have mainly focused on one modality or image-classification accuracy. This narrative review follows the clinical pathway and compares five linked areas: defined diagnostic criteria, sequential digital monitoring, site- and skin-tone-specific interpretation, multimodal imaging, and artificial intelligence (AI). Melanoma and keratinocyte carcinoma are discussed separately because their criteria, diagnostic problems, imaging needs, and management differ. Meta-analyses support dermoscopy over naked-eye examination, although accuracy remains closely related to clinician experience. A randomized trial of three-dimensional total-body photography with sequential dermoscopy led to more procedures and higher costs without finding more malignant tumors over two years. Reflectance confocal microscopy can improve specificity in selected equivocal lesions, while optical coherence technologies add architectural and depth information that is most relevant to keratinocyte carcinoma. AI has moved from retrospective benchmarks to prospective multicenter studies, but false-positive burden, dataset shift, underrepresentation of darker skin tones, limited explainability, and uncertain workflow effects remain. The review also considers foundation models, regulation, and patient-facing tools and uses a SWOT analysis to define research priorities. The field is at an inflection point in evidence, not image quality. Progress should be judged by effects on biopsy decisions, missed-cancer rates, timeliness, cost, access, and patient outcomes in defined populations.

## Simple Summary

Dermoscopy improves the examination of skin lesions by revealing colors and structures that are not visible to the unaided eye. Further progress will depend on more than sharper images. Clinicians need criteria that remain reliable across anatomic sites and skin tones, monitoring strategies that detect meaningful change without driving unnecessary procedures, and additional imaging methods that answer specific clinical questions. Artificial intelligence may support these decisions, but strong performance on selected image collections does not establish safety or benefit in practice. This review examines evidence for established dermoscopic criteria, digital surveillance, site-specific interpretation, multimodal imaging, teledermoscopy, and artificial intelligence. It also identifies practical standards for future studies, including representative populations, prospective validation, clinically relevant comparators, and reporting of false-positive consequences. Progress should be measured by better patient decisions, not solely by image-classification accuracy.

## 1. Introduction

A dermatoscope combines magnification and illumination with reduced surface reflection. Its clinical value extends beyond optics. Dermoscopy reveals colors and structures within the epidermis and superficial dermis that are not visible to the unaided eye. These findings help clinicians decide whether a lesion should be reassured, monitored, biopsied, or treated. In a meta-analysis of studies conducted in clinical settings, dermoscopy had a relative diagnostic odds ratio for melanoma of 15.6 compared with naked-eye examination [1]. A subsequent Cochrane review also found greater diagnostic accuracy when dermoscopy was added to visual inspection, although the size of the benefit varied across study designs and settings [2].

Dermoscopy can be performed using non-polarized and cross-polarized imaging modes. Non-polarized dermoscopy typically uses direct skin contact and a liquid interface, whereas cross-polarized imaging can be performed without contact; the two modes emphasize different structures. Clinicians read the image by naming structures such as pigment network, globules, streaks, dots, vessels, and white or blue–gray areas. They then either recognize the overall pattern or score the features. Established approaches include pattern analysis, the ABCD rule, the Menzies method, the seven-point checklist, and the chaos and clues method. Their performance depends on the criteria used and the experience of the reader [3,4].

Melanoma arises from melanocytes and is the skin cancer most likely to cause death. The main danger is early disease that looks harmless, so dermoscopic diagnosis has to balance two errors: missing an early melanoma and excising too many benign nevi. Some melanomas are small, lack typical structures, or look like nevi, and these are the lesions where false reassurance is most likely. Histopathology remains necessary after biopsy to confirm the diagnosis and characterize invasive disease [1,2].

Basal cell carcinoma (BCC) and cutaneous squamous cell carcinoma (cSCC) arise from keratinocytes and are much more common than melanoma. BCC usually grows locally but can destroy tissue, so the key questions are subtype, depth, and margins. cSCC develops along a continuum from actinic keratosis (AK) to in situ and invasive disease, and a subset of invasive tumors recur or metastasize, especially in immunosuppressed patients. For keratinocyte carcinomas, the diagnostic task is therefore less often to catch a rapidly lethal cancer and more often to separate tumor from benign or inflammatory mimickers, judge invasion, and choose among surgery, destructive or topical treatment, and observation [5,6].

When dermoscopy does not settle the question, two optical methods are often added. Reflectance confocal microscopy (RCM) uses a low-power laser to produce en face (horizontal) grayscale images at cellular resolution, but only to the level of the superficial dermis. Optical coherence tomography (OCT) uses low-coherence near-infrared light to build cross-sectional images. It reaches deeper than RCM, with less cellular detail. Line-field confocal OCT (LC-OCT) combines aspects of both and reaches an intermediate depth of approximately 0.5 mm at near-cellular resolution. It can produce vertical cross-sectional, horizontal, and three-dimensional images [7,8]. Dermoscopy, RCM, and OCT therefore answer different questions: surface pattern, cellular detail, and depth.

Artificial intelligence (AI) in dermoscopy mostly means deep learning, especially convolutional neural networks trained on large sets of labeled images to estimate the probability of a diagnosis such as melanoma or benign nevus [9,10,11]. Newer models, including vision transformers and foundation models, are pretrained on very large and varied image sets, sometimes with clinical data, and then adapted to specific tasks. Performance is usually reported as sensitivity, specificity, and area under the receiver operating characteristic curve, and these values depend strongly on the test set. Evidence also comes in layers: retrospective benchmarks, reader studies, prospective studies, and, rarely, randomized trials of patient outcomes. In this review, AI refers to computational systems that analyze dermoscopic or related image data, alone or with clinical information, to support classification, triage, monitoring, or management decisions.

The practical issue is how dermoscopy can continue to evolve while remaining clinically interpretable. Recent work has made individual structures more measurable, expanded digital surveillance, adapted criteria to anatomic sites where conventional rules perform poorly, paired dermoscopy with optical methods that add cellular or architectural depth, and introduced AI into image interpretation. The benefit of each development depends on how the information is used. A new criterion can increase sensitivity while lowering specificity. A monitoring platform can find subtle changes while increasing biopsies. An algorithm can outperform a reader on a selected dataset and still fail when disease prevalence, image acquisition, or patient characteristics change.

**Figure 1:**
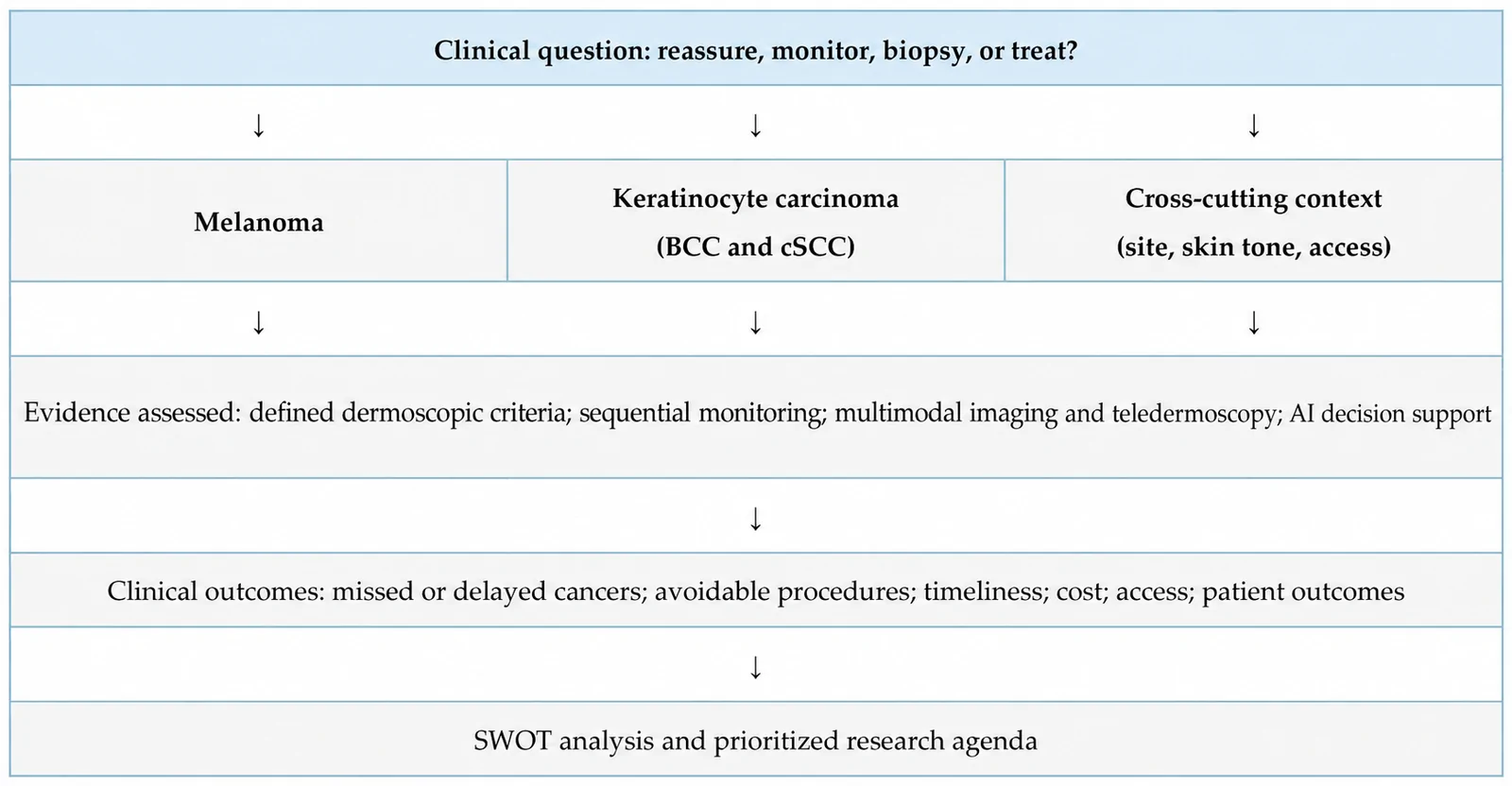
Overview of the review. Melanoma and keratinocyte carcinoma are discussed separately (Section 3 and Section 4). Monitoring, site- and skin-tone-specific interpretation, multimodal imaging, and AI are compared by the clinical decisions they change and are judged against clinical outcomes (Section 8). BCC, basal cell carcinoma; cSCC, cutaneous squamous cell carcinoma; OCT, optical coherence tomography; RCM, reflectance confocal microscopy.

This narrative review examines these developments through the evidence needed for clinical use. Existing reviews tend to address one part of the field. A 2024 systematic review and meta-analysis found AI performance comparable to that of expert dermatologists, but only 3 of 53 included studies were prospective [12]. Another meta-analysis examined human–AI interaction [13], while other reviews have cataloged computer-vision models, datasets, and validation problems [11]. Modality-specific reviews have addressed noninvasive technologies for cSCC [6], LC-OCT [14], BCC subtyping [8], and dermoscopy in skin of color [15]. The present review adds four elements. It places diagnostic criteria, monitoring, site-specific interpretation, multimodal imaging, teledermoscopy, and AI within one clinical pathway (Figure 1). It discusses melanoma and keratinocyte carcinoma separately because they involve different diagnostic questions, imaging needs, and management choices. It evaluates where each method enters care, which decision it changes, and whether that change improves outcomes. It also summarizes evidence gaps across skin tones and anatomic sites, reviews recent work on explainability, foundation models, regulation, and patient-facing tools, and ends with a SWOT analysis and prioritized research agenda.

## 2. Scope and Literature Identification

We used a clinical-translation framework to organize this narrative review. The framework emphasizes diagnostic accuracy, workflow effects, generalizability, and patient outcomes.

Databases and search period. We searched PubMed/MEDLINE and added backward citation searching and targeted searches of publisher and regulatory websites. The search included the literature available through 30 September 2026. Literature search and evidence synthesis were carried out by D.N.P., W.H.C., and J.C.

*Search terms*. Search concepts combined dermoscopy or dermatoscopy with melanoma, basal cell carcinoma, squamous cell carcinoma, sequential digital monitoring, total-body photography, acral, facial, nail, mucosal, skin of color, reflectance confocal microscopy, optical coherence tomography, teledermoscopy, artificial intelligence, deep learning, explainability, regulation, and implementation.

Selection criteria. We prioritized systematic reviews and meta-analyses, randomized or prospective clinical studies, multicenter validation studies, expert guidance, and recent work addressing generalizability, equity, or workflow. Evidence was included when it directly informed a clinical decision or a limitation of translation. Highly selected technical studies without clinically relevant validation were used only to illustrate the benchmark-to-bedside gap. This was a critical narrative synthesis, not an exhaustive evidence inventory. It was not preregistered, and no PRISMA flow diagram or quantitative pooling was performed.

## 3. Melanoma: From Expert Pattern Recognition to Defined Criteria

Melanoma and keratinocyte carcinoma are considered separately because their dermoscopic criteria, diagnostic challenges, use of additional imaging, and management differ (Table 1). Section 3 addresses melanoma and sequential monitoring, which has primarily focused on melanoma in the studies reviewed here. Section 4 addresses BCC and cSCC.

### 3.1. Diagnostic Accuracy and the Role of the Observer

Dermoscopy improves visual examination, but its performance remains closely tied to training and case selection. A 2025 systematic review and meta-analysis of 100 studies found that experienced dermatologists using dermoscopy or dermoscopic images had sensitivity and specificity of 85.7% and 81.3% for melanoma. The corresponding estimates were 78.0% and 69.5% for inexperienced dermatologists and 49.5% and 91.3% for primary care physicians [3]. Experienced dermatologists had 5.7-fold higher odds of accurate melanoma diagnosis with dermoscopy than with clinical examination alone. These findings support the use of dermoscopy and show why accuracy reported for a device or study setting cannot be assumed for every user.

The maturation of dermoscopy has depended on converting gestalt into observable features. In a systematic review of 40 studies including 22,796 lesions and 5736 melanomas, pseudopods and shiny white structures were highly specific for melanoma, whereas irregular pigmentation and blue–white veil were more sensitive but less specific [4]. No single structure is sufficient in every lesion. The value of a criterion depends on how it changes the probability of disease in the clinical context, and combinations of structures generally matter more than isolated findings. Reader experience, lesion spectrum, diagnostic threshold, and intended decision all affect how a pooled accuracy measure should be interpreted.

### 3.2. Sequential Digital Monitoring

Sequential digital dermoscopy imaging extends interpretation across time. Its main use is not to confirm an already classic melanoma, but to identify lesions that are equivocal at baseline and subsequently change. In a case–control analysis of 206 melanomas, only 40.8% of melanomas detected during digital monitoring were recognized from a single follow-up image, compared with 78.6% of melanomas diagnosed at baseline [19]. Among monitored melanomas, 59.2% had not developed conventional melanoma-specific criteria and were detected only by side-by-side comparison. This finding explains why temporal context can contribute diagnostic information that a static algorithm may miss.

Monitoring can also lead to additional procedures. In a 2025 randomized trial of 315 adults at high risk for melanoma, adding three-dimensional total-body photography, sequential digital dermoscopy, and teledermatology review every six months resulted in more excisions or biopsies than usual care [22]. A prespecified economic analysis found higher costs and similar numbers of malignant tumors detected over two years [23]. The finding challenges the assumption that an intensive surveillance package improves outcomes simply because it records more change. It does not resolve the value of sequential monitoring itself because the intervention combined several components and relied on junior-clinician examinations with specialist review. Patient selection, reviewer expertise, visit frequency, and biopsy thresholds may explain part of the added procedure burden. Monitoring studies are most informative when they report the number needed to excise, interval cancers, melanoma thickness, adherence, patient burden, and costs alongside the number of changing lesions found.

The patients most likely to derive net benefit are those in whom baseline excision of every atypical lesion would be impractical and longitudinal comparison can resolve uncertainty. International Dermoscopy Society consensus supports digital monitoring for patients with at least 60 nevi or a CDKN2A mutation, and for selected patients with more than 40 nevi plus a personal history of melanoma, red hair or an MC1R mutation, or organ transplantation [24]. A rapidly changing nodular lesion or a lesion that already meets a biopsy threshold should not be delayed for monitoring. Follow-up reliability also matters because a surveillance strategy loses value when interval images cannot be obtained or acted upon. Because the randomized trial already enrolled adults at high risk for melanoma [22], “high risk” alone may not be enough to select patients for intensive monitoring. Trials that apply the International Dermoscopy Society criteria, report outcomes by risk group, and compare monitoring intensities would be more informative.

## 4. Keratinocyte Carcinomas and the Larger Diagnostic Burden

BCC and cSCC account for a large share of the lesions evaluated in routine practice. Dermoscopic clues for BCC include arborizing vessels, short fine telangiectasias, blue–gray ovoid nests, maple leaf-like areas, spoke-wheel areas, ulceration, and shiny white structures. A 2019 systematic review and meta-analysis reported pooled sensitivity of 91.2% and specificity of 95.0% for BCC [5]. In directly comparative studies, adding dermoscopy to naked-eye examination increased sensitivity from 66.9% to 85.0% and specificity from 97.2% to 98.2%. Performance was better for pigmented than nonpigmented tumors and for expert, in-person assessment. A 2025 systematic review further emphasized the wider range of vascular, white, yellow, blue, pigmented, and erosive features now associated with BCC, while noting persistent difficulty with superficial tumors on the trunk and extremities [18].

For actinic keratosis and cSCC, the dermoscopic vocabulary includes scale, keratin, white circles, blood spots, ulceration, and polymorphous or hairpin vessels. These features overlap across a biologic continuum and can change with lesion thickness, inflammation, and anatomic site. Evidence for advanced noninvasive imaging in cSCC is less mature than the evidence for melanoma. A 2024 systematic review and meta-analysis of noninvasive technologies included 15 studies, with only seven eligible for quantitative synthesis. Reported sensitivities were 92% for high-frequency ultrasound, 75% for OCT, and 63% for RCM, with corresponding specificities of 88%, 95%, and 96% [6]. The small numbers, heterogeneous criteria, and dependence on operator interpretation limit broad conclusions. For keratinocyte cancers, a clinically useful advance would distinguish tumors requiring biopsy or surgery from lesions suitable for noninvasive treatment or observation. Histopathology remains the reference standard when subtype or invasion affects management.

Management pathways also differ from melanoma. For melanoma, a suspicious dermoscopic impression usually leads to excision-based diagnosis, and histologic thickness then determines margins and staging. For keratinocyte carcinoma, the same impression may lead to surgery, Mohs surgery, destructive or topical treatment, or observation, depending on subtype, depth, site, and patient factors. For this reason, imaging that estimates depth or subtype is particularly relevant to keratinocyte carcinoma, whereas in melanoma its main role has been to improve selection for biopsy [7,20].

## 5. Site-Specific Dermoscopy and Representation

### 5.1. Acral, Facial, Mucosal, and Nail-Unit Lesions

Interpretation changes by anatomic site. On acral skin, pigment is assessed in relation to skin ridges. Facial assessment centers on follicular openings, while nail-unit assessment focuses on the pattern of longitudinal bands. Mucosal lesions are evaluated by their colors and structureless areas. Table 2 summarizes these site-specific reading frames and their main pitfalls.

Most general dermoscopic algorithms assume the architecture of nonacral skin. On palms and soles, pigment distribution relative to dermatoglyphics is central. The parallel ridge pattern remains a major clue to acral melanoma, whereas parallel furrow and lattice-like patterns more often support a benign lesion. In a large reader study validating the BRAAFF checklist, 175 readers evaluated 533 acral nevi and 144 acral melanomas. The algorithm achieved sensitivity of 92.5% and specificity of 65.0%, but it did not outperform unaided pattern recognition [16]. The checklist can support standardization and teaching, while interpretation still depends on the observer’s experience.

Facial lesions require a different frame because follicles interrupt the pigment network. Gray dots around follicles, asymmetric pigmented follicular openings, rhomboidal structures, and progressive follicular obliteration can raise concern for lentigo maligna, but photodamage, pigmented actinic keratosis, and other benign or premalignant lesions create substantial overlap [17,25]. Nail-unit assessment instead centers on longitudinal bands, including width, color variation, irregularity of lines, periungual pigmentation, nail dystrophy, and documented change. These findings must be interpreted with the patient’s age, involved digit, trauma, and ethnic melanonychia in mind, and suspicious lesions still require an appropriately planned nail-matrix biopsy [17,26]. Mucosal lesions are a separate low-prevalence, high-consequence setting. Structureless areas, multiple colors, and blue, gray, or white components can be concerning, but the evidence is dominated by small series and standardized criteria remain limited [17,27]. Across these sites, histopathology remains necessary when noninvasive findings cannot safely resolve uncertainty. Site-specific validation also needs early lesions, benign mimickers, and nonclassic presentations rather than only image-selected examples.

### 5.2. Skin Tone and Diagnostic Equity

The dermoscopy literature has not represented skin tones evenly. An International Dermoscopy Society systematic review found that published analyses in skin of color were generally small and concentrated in only part of the darker-phototype spectrum [15]. A separate scoping review likewise identified limited representation of skin of color in studies designed to distinguish benign from malignant lesions [28]. This gap affects both clinician education and AI development because reference images, feature prevalence, and background pigmentation can influence interpretation.

Consensus work has adapted the International Dermoscopy Society’s descriptive parameters for phototypes IV to VI, but these criteria still require outcome-based validation [29]. The available literature is largely descriptive, uses inconsistent measures of skin tone, and rarely reports sensitivity, specificity, or diagnostic failure separately across phototypes. As a result, it is difficult to determine whether apparent feature differences arise from pigmentation, lesion mix, anatomic site, image acquisition, or sampling. Useful equity analyses should define and preferably measure skin tone, prespecify subgroup estimates with uncertainty intervals, and adequately represent uncommon malignancies and special sites. Broad racial or ethnic categories should not be used as biological substitutes for pigmentation. Access to specialty review, biopsy follow-up, and adequate image quality also matters because an accurate tool can widen disparities if it is available only where dermatology resources are already concentrated.

Taken together, the main evidence gaps are: small samples concentrated in part of phototypes IV to VI; no consistent way of describing skin tone; few subgroup estimates of sensitivity, specificity, or diagnostic failure; limited data on uncommon malignancies and special sites; and little information on access and follow-up. The same pattern appears in AI research. In PROVE-AI, 96% of participants were White [30], and a diverse, curated clinical image set showed lower AI performance on darker skin tones [31] (see Section 7.2 and Section 7.3).

## 6. Dermoscopy as One Layer of a Multimodal System

### 6.1. Reflectance Confocal Microscopy

RCM provides en face, cellular-resolution images of the epidermis and superficial dermis. Its strongest clinical role is often a second-level test for a lesion that remains equivocal after clinical and dermoscopic assessment. In a randomized clinical trial of suspect lesions, adding RCM increased positive predictive value from 18.9% to 33.3%, reduced the benign-to-malignant excision ratio from 3.7:1 to 1.8:1, and reduced the number needed to excise by 43.4% [7]. A Cochrane review also found that RCM could improve specificity in selected equivocal lesions, while noting variation in study quality, thresholds, and populations [20].

These findings support selective use of RCM after clinical and dermoscopic assessment. RCM requires equipment, acquisition time, training, and a lesion that can be imaged and interpreted. It is most useful when the pretest probability warrants further evaluation but uncertainty remains high enough to avoid immediate biopsy. Studies define its role more clearly when they report where RCM enters the pathway and how often it changes management, in addition to image-classification accuracy.

### 6.2. Optical Coherence Technologies

OCT adds cross-sectional architectural information at greater depth than dermoscopy, although with lower cellular resolution than RCM. Line-field confocal OCT (LC-OCT) combines aspects of both approaches, offering near-cellular resolution at an intermediate depth of approximately 0.5 mm and providing vertical cross-sectional, horizontal, and three-dimensional imaging [8,14]. A 2024 meta-analysis of five LC-OCT studies, comprising 904 lesion-level analyses, estimated pooled sensitivity of 86.9% and specificity of 91.1% for malignant skin tumors [14]. The evidence base remains modest and includes different tumor types, readers, and diagnostic criteria. A 2025 systematic review of noninvasive imaging for BCC subtyping included 19 studies and 2650 lesions. One included study reported dermoscopic sensitivity and specificity of 81.9% and 81.8% for superficial BCC, while LC-OCT and high-definition OCT produced strong results in selected subtype analyses [8]. Heterogeneous protocols and small subgroup samples prevent these values from being treated as universal performance estimates.

The most defensible use of optical imaging is tied to a concrete question, such as whether a lesion is likely to be superficial, whether an equivocal area warrants biopsy, or whether clinically visible margins underestimate disease extent. An exploratory study in this Special Issue illustrates another possibility by combining dermoscopy with noninvasive imaging to examine vascular change in BCC before and after topical imiquimod [21]. Such mechanistic or monitoring applications are valuable when the imaging endpoint is prespecified and linked to a clinical or biologic outcome.

The modalities answer different questions. Dermoscopy is the first-line method for lesion diagnosis, and RCM can add cellular detail to improve specificity in selected equivocal lesions [7,20]. OCT and LC-OCT provide cross-sectional information about tissue architecture that surface imaging cannot show [8,14]. OCT, LC-OCT, and high-frequency ultrasound in cSCC [6] may also help estimate whether a lesion is superficial or deeper. These depth data come from small, heterogeneous studies, and histopathology remains necessary when invasion determines treatment. In keratinocyte carcinoma, the clinically useful question is whether imaging changes the need, location, or timing of biopsy, clarifies a treatment boundary, or supports a less invasive pathway without compromising safety. Monitoring response to topical therapy is another possible use [21].

### 6.3. Teledermoscopy

Teledermoscopy can move image acquisition closer to primary care while preserving specialist interpretation. A 2024 systematic review of teledermatology for nonmelanoma skin cancer found broadly comparable, though often lower, diagnostic accuracy than face-to-face assessment and identified image quality, clinical history, and inclusion of dermoscopic images as important determinants of performance [32]. Shorter time to specialist input can be meaningful in underserved settings, but triage gains do not guarantee shorter time to biopsy or treatment if downstream capacity is limited.

Teledermoscopy changes the pathway as well as the location of review. The person capturing the image, the available clinical history, and responsibility for follow-up may all differ from an in-person visit. Evaluation should include image failure, time to expert review and definitive diagnosis, escalation to in-person care, and loss to follow-up.

## 7. Artificial Intelligence: From Benchmark Accuracy to Clinical Usefulness

### 7.1. What Reader Studies Established

Early deep neural networks showed that image classification could reach dermatologist-level performance under experimental conditions. Esteva and colleagues trained one network across a broad taxonomy of skin lesions and reported dermatologist-comparable discrimination on selected classification tasks [9]. Haenssle and colleagues later compared a convolutional neural network with 58 dermatologists and found higher specificity for the network at matched sensitivity [10]. These studies established technical feasibility. Their designs did not determine how an algorithm should be incorporated into an examination, how it performs on lesions excluded from training, or whether its recommendations reduce missed cancers or unnecessary procedures.

Human–computer collaboration studies shifted attention from competition to decision support. Tschandl and colleagues found that algorithmic support could improve reader performance, with gains that varied by clinician experience and by how advice was presented [33]. A 2024 systematic review and meta-analysis of 12 studies comprising 67,700 diagnostic evaluations found that algorithmic support improved pooled sensitivity and specificity across clinician groups, with the largest gains among non-dermatologists [13]. This pattern is clinically plausible: decision support may be most useful when uncertainty is moderate, while incorrect or overconfident output can pull a reader away from a correct initial impression. Useful evaluations compare unaided and aided decisions, stratify by reader experience, examine disagreement cases, and show when clinicians accept or reject the recommendation.

### 7.2. Prospective Validation and the Sensitivity-Specificity Tradeoff

Prospective studies have begun to narrow the gap between benchmark performance and clinical use. In the PROVE-AI study, 435 participants contributed 603 lesions, including 95 melanomas. At a prespecified threshold targeting 95% sensitivity, the open-source algorithm achieved sensitivity of 96.8% and specificity of 37.4% [30]. Put clinically, roughly six of every ten nonmelanoma lesions would be assigned a positive result at that threshold. If the output were treated as an automatic biopsy trigger, the false-positive workload would be substantial; if it were used only as one input to clinician judgment, the effect would depend on when clinicians accept, override, or disregard the score. Dermatologists’ discrimination improved after exposure to the algorithm score, but 96% of participants were White, limiting conclusions about broader populations. The study is valuable precisely because it makes the sensitivity-specificity tradeoff and the downstream decision explicit.

A 2024 prospective multicenter study evaluated an open-source ensemble on a heterogeneous set collected across eight German university hospitals, four camera configurations, rare subtypes, and special sites. The algorithm had higher balanced accuracy and sensitivity than dermatologists but lower specificity [34]. In a separate prospective primary-care trial across 36 Swedish centers, an AI decision-support application achieved high discrimination in routine use. Its performance still needs to be interpreted within the care pathway and disease prevalence for which it was tested [35]. These studies provide stronger evidence than retrospective image challenges, but randomized evaluations are still needed to measure effects on patient management and safety.

### 7.3. Dataset Shift, Underrepresentation, and Failure Analysis

Image datasets vary by institution, camera, disease prevalence, referral threshold, and confirmation method. In the 2019 International Skin Imaging Collaboration challenge analysis, the best algorithm’s balanced accuracy fell from 82.0% on a familiar benchmark distribution to 58.8% on a dataset designed to better reflect clinical variability [36]. Diagnoses absent from training were particularly difficult, and image artifacts affected performance in diagnosis-dependent ways. These limitations have direct clinical consequences. A system that cannot recognize an unfamiliar input may produce a confident answer when deferral would be safer.

Representation also affects performance. Daneshjou and colleagues evaluated dermatology AI on a pathologically confirmed, diverse clinical image set and found lower performance on darker skin tones and uncommon diseases; targeted fine-tuning reduced the gap [31]. The finding supports deliberate dataset design while showing that demographic balance alone does not resolve bias. Clinical images, dermoscopic images, lesion types, disease prevalence, and confirmation standards all require attention. Subgroup analyses should be prespecified and reported with uncertainty rather than as a single aggregate score.

### 7.4. Explainability, Multimodal Models, Regulation, and Patient-Facing Tools

Small models evaluated only on internal data can report high performance while leaving the clinical question unanswered. For example, a study using 1000 images from a public dataset reported 92% accuracy, precision, sensitivity, specificity, and F1 score after a random train–validation–test split [37]. The result shows technical feasibility but does not address external validation, prospective image acquisition, disease prevalence, or the consequences of a false result.

Explainability is often proposed as a bridge between model output and clinical trust. Saliency maps and related visual explanations may reveal that a model is attending to an artifact or background feature, while concept-based explanations can express outputs in terms that are closer to dermoscopic reasoning. They do not, however, prove that the model reached its result for a clinically valid reason. A systematic review identified 37 studies applying explainable AI to skin-cancer images, but only three evaluated how explanations affected human performance or confidence [38]. Human-in-the-loop evaluation should therefore move beyond aided-versus-unaided accuracy. It should test calibrated uncertainty, the ability to defer unfamiliar inputs, the handling of clinician–algorithm disagreement, override behavior, and escalation pathways when the model or clinician is uncertain.

Foundation and multimodal models extend the task beyond classification of a single cropped lesion. PanDerm was pretrained on more than two million images from 11 institutions across total-body photography, clinical photography, dermoscopy, and dermatopathology and was evaluated across several diagnostic, monitoring, and prognostic tasks [39]. This scale and flexibility may support longitudinal comparison and integration of different image types. Clinical benefit still requires prospective evidence showing which input combinations improve a decision, how performance changes across sites and populations, and whether the model remains calibrated after deployment.

Regulation follows the intended use and risk of a system. In the United States, an AI-enabled system that provides diagnostic information is regulated as a medical device (software as a medical device, SaMD), with requirements for premarket evidence and management of changes across the product life cycle [40]. In the European Union, AI-enabled medical-device software may fall under both medical-device rules and the EU AI Act, depending on its intended use and risk [41]. The manufacturer’s claimed use therefore affects the evidence required. A clinician-facing second-read tool, an autonomous triage system, and a patient-facing smartphone application create different risks and oversight needs. A 2026 European review found that dermatology apps were widely available but often lacked strong clinical validation, transparent regulatory status, or evidence across diverse users [42]. Patient-facing tools should report failed image capture, false reassurance, false alarms, time to clinical evaluation, privacy, and access to follow-up in addition to accuracy.

## 8. Standards for the Next Generation of Dermoscopy Studies

The next phase of dermoscopy research should be judged at the level of clinical decision. A study of a new criterion, imaging device, monitoring platform, or algorithm should state who will use it, in which patients, at what point in care, and what action follows the result. The comparator should be current practice in the same setting. Histopathology remains the appropriate reference for excised lesions, but follow-up, expert consensus, and mixed reference standards must be reported transparently when universal biopsy would be unethical or impractical.

Accuracy is necessary but incomplete. Sensitivity and specificity should be accompanied by predictive values at the observed prevalence, calibration, indeterminate or failed-test rates, and performance across relevant sites and skin tones. Decision outcomes should include benign-to-malignant excision ratio, number needed to excise, missed or delayed cancers, tumor thickness, time to diagnosis, cost, patient burden, and clinician workload. For AI, external and prospective validation should precede claims of clinical readiness, and post-deployment monitoring should address drift, device changes, and uncommon inputs. Early live clinical evaluations should also follow DECIDE-AI, which covers clinical performance, safety, human factors, and workflow integration [43]. Table 3 summarizes priorities for evaluating these tools. Table 4 shows where each approach may fit in care and which evidence is still missing.

### SWOT Analysis of Clinical Translation

The SWOT analysis in Table 5 shows where technical progress may support care and where translation can fail.

These categories lead to practical study priorities. Expected strengths should be tested in the settings where they are intended to help. Weaknesses should be prespecified as failure modes, opportunities should be tied to measurable changes in care, and threats should be monitored after deployment. Clinicians interviewed about AI in skin-cancer care emphasized transparency, validation, usability, access, regulation, and clear responsibility [44]. Implementation research should also examine training requirements, reimbursement for acquisition and interpretation, responsibility for missed or unnecessary procedures, and whether frequent reliance on automated output changes clinicians’ unaided diagnostic performance over time.

## 9. Conclusions and Future Directions

Dermoscopy is at an inflection point in how evidence is judged. Defined criteria make interpretation more teachable, sequential monitoring adds change over time, and site-specific frameworks account for anatomic exceptions. RCM and OCT can answer cellular, architectural, or depth-related questions that surface imaging cannot. AI can organize or extend these inputs, but its value remains dependent on the intended population, user, threshold, workflow, and consequences of error.

The immediate research agenda requires clear priorities. Monitoring studies should first identify which high-risk populations gain earlier diagnosis without an unacceptable increase in procedures or cost. Multimodal studies should then define the sequence in which dermoscopy, RCM, OCT, teledermoscopy, or AI changes a decision instead of testing each tool against histopathology in isolation. AI evaluations should move from selected image sets to prospective care pathways that report failed acquisition, deferral, clinician override, subgroup performance, false-positive burden, and patient-level outcomes.

Implementation should be evaluated with the same rigor as diagnostic accuracy. Training must specify who acquires and interprets images. Reimbursement should reflect demonstrated clinical value rather than the presence of a new technology. Liability must be clear when a clinician and an algorithm disagree, and post-deployment monitoring should detect drift or unintended changes in biopsy thresholds. Preserving clinician skill is also a safety outcome; decision support should make uncertainty visible and support escalation rather than replace examination or judgment.

A meaningful inflection point will occur when technical improvements lead to better decisions: fewer missed or delayed cancers, fewer avoidable procedures, timely access to expertise, acceptable costs, and benefit across the patients who will use the system.

## Figures and Tables

**Table 1:** Melanoma and keratinocyte carcinoma compared across the diagnostic and management pathway.

Feature	Melanoma	Keratinocyte Carcinoma (BCC and cSCC)
**Typical dermoscopic criteria**	Pseudopods and shiny white structures (high specificity); irregular pigmentation and blue–white veil (more sensitive, less specific); combinations matter more than single findings [4]. Criteria change by site [16,17].	BCC: arborizing vessels, blue–gray ovoid nests, maple leaf-like and spoke-wheel areas, ulceration, shiny white structures [5,18]. AK and cSCC: scale, keratin, white circles, blood spots, polymorphous or hairpin vessels.
**Main diagnostic challenge**	Early or featureless melanomas and nevus-like lesions; risk of false reassurance. In one monitoring series, 59.2% of monitored melanomas lacked conventional melanoma-specific criteria [19].	Nonpigmented and superficial tumors; overlap along the AK-cSCC continuum; judging thickness and invasion [5,6].
**Role of added imaging**	Mainly second-level testing and monitoring of equivocal lesions, to improve specificity and reduce unnecessary excisions [7,19,20].	OCT, LC-OCT, and high-frequency ultrasound for subtype, depth, and architecture; evidence is still limited [6,8,14].
**Management pathway**	Biopsy or excision confirms the diagnosis; histologic thickness then guides margins and further staging.	Surgery, Mohs surgery, destructive or topical therapy, or observation, chosen by subtype, depth, site, and patient factors; histopathology still needed when invasion changes management [6,8,21].

AK, actinic keratosis; BCC, basal cell carcinoma; cSCC, cutaneous squamous cell carcinoma; LC-OCT, line-field confocal optical coherence tomography; OCT, optical coherence tomography.

**Table 2:** Site-specific dermoscopic interpretation: how the reading frame and pitfalls differ.

Site	How Interpretation Differs	Main Pitfalls	Refs
**Acral (palms and soles)**	Pigment distribution relative to dermatoglyphics; parallel ridge pattern suggests melanoma, parallel furrow and lattice-like patterns suggest a benign lesion.	A validated checklist (BRAAFF) did not outperform unaided pattern recognition, so interpretation still depends on experience.	[16,17]
**Face**	Follicles interrupt the pigment network; gray dots around follicles, asymmetric pigmented follicular openings, rhomboidal structures, and progressive follicular obliteration raise concern for lentigo maligna.	Overlap with photodamage, pigmented actinic keratosis, and other benign or premalignant lesions.	[17,25]
**Nail unit**	Longitudinal bands: width, color variation, line irregularity, periungual pigmentation, nail dystrophy, and documented change.	Must be read with age, digit, trauma, and ethnic melanonychia; suspicious bands still need a planned nail-matrix biopsy.	[17,26]
**Mucosa**	Structureless areas, multiple colors, and blue, gray, or white components.	Low prevalence, high consequence; evidence from small series; few standardized criteria.	[17,27]

Across all sites, histopathology remains necessary when noninvasive findings cannot safely resolve uncertainty.

**Table 3:** Practical evaluation priorities for studies of emerging dermoscopy tools.

Domain	Minimum Comparator	Clinically Useful Outcomes	Common Failure to Avoid
**New dermoscopic criterion**	Current trained-reader assessment	Sensitivity, specificity, interobserver agreement, biopsy decisions	Image-selected case–control testing without a clinical threshold
**Digital monitoring**	Risk-matched usual surveillance	Number needed to excise, interval melanoma, thickness, adherence, cost	Counting detected changes without measuring added procedures
**RCM or OCT**	Dermoscopy-guided standard care	Management change, avoided biopsy, missed malignancy, time and resource use	Reporting stand-alone image accuracy outside the intended pathway
**Teledermoscopy**	Usual referral pathway	Image failure, time to expert decision, time to diagnosis, loss to follow-up	Measuring triage speed while ignoring downstream delay
**Artificial intelligence**	Unaided and aided clinicians in the deployment setting	Calibration, subgroup performance, biopsy impact, override behavior, safety	Treating retrospective benchmark superiority as clinical benefit

OCT, optical coherence tomography; RCM, reflectance confocal microscopy.

**Table 4:** Clinical roles and unresolved evidence needs for emerging dermoscopy approaches.

Approach	Clinical Decision Supported	Best-Supported Contribution	Main Unresolved Evidence Need
**Defined dermoscopic criteria** [3,4,15,16,29]	Standardize lesion interpretation across readers and anatomic sites	Convert visual patterns into reproducible features that support training	Prospective validation across experience levels, anatomic sites, and skin tones
**Sequential digital monitoring** [19,20,21,22,23]	Determine whether an equivocal lesion has changed enough to biopsy	Add temporal information when classic melanoma criteria are absent	Risk-stratified trials reporting excision efficiency, interval melanoma, thickness, patient burden, and cost
**RCM** [7,20]	Clarify an equivocal lesion before biopsy	Improve specificity and may reduce unnecessary excision	Prospective pathway studies reporting avoided biopsy, missed malignancy, access, time, and cost
**OCT or LC-OCT** [8,14]	Assess lesion architecture and depth, especially in keratinocyte cancer	Add structural information beyond surface imaging	Multicenter validation against histopathology with management outcomes
**Teledermoscopy** [32]	Extend expert review and triage to remote settings	May shorten access to specialist interpretation	Comparative pathway studies reporting image failure, time to diagnosis, and completion of care
**AI-assisted diagnosis** [12,33,43]	Support lesion classification and clinician decisions	May improve diagnostic performance, especially for less-experienced clinicians	Prospective and randomized studies reporting biopsy impact, safety, subgroup performance, and workflow

AI, artificial intelligence; LC-OCT, line-field confocal optical coherence tomography; OCT, optical coherence tomography; RCM, reflectance confocal microscopy.

**Table 5:** SWOT analysis of the clinical translation of dermoscopy and related tools.

**Strengths**	**Weaknesses**
Portability and low cost of dermoscopy Established diagnostic vocabulary Ability to document change over time Compatibility with teledermoscopy, RCM, OCT, and decision support	Operator dependence Variable image acquisition Limited depth information Heterogeneous reference standards Uneven representation across skin tones and special sites
**Opportunities**	**Threats**
Risk-adapted surveillance More selective use of second-level imaging Multimodal models that combine longitudinal and clinical information Remote specialist support in underserved settings	False-positive cascades and false reassurance Automation bias and loss of clinician skill through overreliance Inequitable access and privacy concerns Unresolved reimbursement, liability, and regulatory responsibilities

SWOT, strengths, weaknesses, opportunities, and threats; OCT, optical coherence tomography; RCM, reflectance confocal microscopy.

## Data Availability

No new data were created or analyzed in this study. Data sharing is not applicable to this article.
